# The Bright and Dark Sides of Cognitive Reserve: Insights from Middle‐Aged Adults at High Risk for Alzheimer's Disease

**DOI:** 10.1002/alz70857_102767

**Published:** 2025-12-25

**Authors:** Yonatan M Schwartz, Michal S Beeri, Sharon Naparstek

**Affiliations:** ^1^ Bar‐Ilan University, Ramat Gan, Israel; ^2^ Herbert and Jacqueline Krieger Klein Alzheimer's Research Center at Rutgers Brain Health Institute, New Brunswick, NJ, USA

## Abstract

**Background:**

The Cognitive Reserve (CR) theory posits that intellectual enrichment, such as education, enhances brain efficiency, enabling individuals to withstand more neurodegeneration and delay cognitive decline. However, CR does not slow disease progression, potentially leading to a faster decline once impairment begins. This study examined CR's impact on cognitive performance over time in middle‐aged adults at increased risk for Alzheimer's Disease (AD).

**Method:**

Two hundred and twenty‐five middle‐aged adults (129 females; ages 40–66 years, M=54.8, SD=6.8; education 11‐27 years, M=16.5, SD=3.02) with AD parental history from the Israel Registry for Alzheimer's Prevention study underwent baseline cognitive assessments and brain MRI scans, followed by a cognitive reassessment 2.5–11.2 years later (M=6.5, SD=2.1). CR was measured by baseline vocabulary, and cognitive function was evaluated across working memory, episodic memory, attention/processing speed, and reasoning/problem‐solving. Mediation analyses assessed baseline cognitive function as a mediator of follow‐up outcomes, controlling for sex, age, education, intracranial volume, brain atrophy, and the time interval between assessments.

**Result:**

Higher CR was positively associated with baseline performance in working memory (β = .16, *p* < .01), episodic memory (β = .25, *p* < .001), attention/processing speed (β = .34, *p* < .001), and reasoning/problem‐solving (β = .45, *p* < .001), with similar indirect associations at follow‐up (β = .11, *p* < .01 to β = .35, *p* < .001), mediated by baseline performance. However, higher CR predicted greater cognitive decline in working memory (β=‐.17, *p* < .02), episodic memory (β=‐.12, *p* < .001), attention/processing speed (β=‐.17, *p* < .001), and reasoning/problem‐solving (β=‐.11, *p* < .01), with these effects mediated by baseline performance except in working memory, where the effect was largely direct.

**Conclusion:**

These findings suggest that CR compensates for the functional decline caused by brain atrophy but does not delay its progression. This compensatory effect is evident in its ability to maintain cognitive performance and postpone decline temporarily. However, once cognitive decline begins, it tends to occur more rapidly. These results underscore the need for early interventions targeting the root causes of cognitive deterioration to reduce the long‐term impact of disease progression.

